# *Uwhangchungsimwon*, A Standardized Herbal Drug, Exerts an Anti-Depressive Effect in a Social Isolation Stress-Induced Mouse Model

**DOI:** 10.3389/fphar.2019.01674

**Published:** 2020-01-31

**Authors:** Hyeon-Muk Oh, Jin-Seok Lee, Seo-Woo Kim, Young-Taeck Oh, Won-Yong Kim, Sung-Bae Lee, Yong-Rae Cho, Yoo-Jin Jeon, Jung-Hyo Cho, Chang-Gue Son

**Affiliations:** ^1^ College of Korean Medicine, Daejeon University, Daejeon, South Korea; ^2^ Liver and Immunology Research Center, Daejeon Korean Medicine Hospital of Daejeon University, Daejeon, South Korea

**Keywords:** herbal medicine, *Uwhangchungsimwon*, depression, 5-HT, brain-derived neurotrophic factor

## Abstract

**Introduction:**

*Uwhangchungsimwon* (UCW) is one of the most representative standardized herbal drugs for the treatment of central nervous system diseases, including mood disorders, and has been used for over 600 years in Korea and China. In spite of the long clinical application of UCW, no experimental evidence for its use against depressive disorders exists. Here, we performed an animal study to investigate the anti-depressive effect of UCW and the underlying mechanisms.

**Methods:**

A social isolation-induced depressive-like model was produced using C57BL/6J male mice by housing the mice individually for 31 days, and the mice underwent daily oral administration of distilled water, UCW (100, 200, 400 mg/kg) or fluoxetine (20 mg/kg) during the final 17 days. A tail suspension test (TST), forced swimming test (FST), and open field test (OFT) were used to explore the effects of UCW on depressive-like behaviors. 5-Hydroxytryptamine (5-HT) was measured in the dorsal raphe nuclei (DRN) using immunofluorescence. The serum corticosterone level was measured with its receptor and catecholamine, along with cAMP response element-binding protein (CREB) and brain-derived neurotrophic factor (BDNF) in the hippocampus.

**Results:**

Social isolation stress effectively induced depressive-like behaviors, and UCW treatment significantly improved the symptoms of depressive-like behavior in the FST, TST, and OFT. The isolation stress-induced depletion of 5-HT was significantly ameliorated by UCW treatment. UCW also attenuated the activation of the glucocorticoid receptor (GR) and the elevated serum corticosterone level, as well as the hippocampal levels of dopamine and norepinephrine. Dexametasone-derived translocation of GR was inhibited by UCW treatment in PC12 cells and HT22 cells. In addition, alterations of tryptophan hydroxylase 2 (TPH2), BDNF, and CREB in the protein analyses were notably regulated by UCW treatment.

**Conclusions:**

These results provide animal-based evidence for the anti-depressive effect of UCW, and its underlying mechanisms may involve regulating the serotonergic system, the hypothalamic-pituitary-adrenal (HPA) axis, and neurotrophin.

## Introduction

Depression (a major depressive disorder) is a life-threatening psychiatric disorder that presents symptoms of depressed or irritable mood, diminished interest in usual activities, loss of appetite, and even high risk of suicide (Nizamie and Garg, 2014). The prevalence of depression was estimated at 4.4% in 2015, corresponding to approximately 322 million people worldwide. In addition, the estimated number of people suffering from depression between 2005 and 2015 increased by 18.4% (World Health Organization, 2017). However, because of the multiple and complicated pathogenic factors involved in depression, the current antidepressants exhibit a narrow spectrum of activity and the limited effect (approximately 47% response rate and 28% remission rate) in the course of treatment among users (Gaynes et al., 2009; Duman et al., 2016).

The most widely used antidepressants are selective serotonin reuptake inhibitors (SSRIs), which maintain the levels of serotonin at post-synaptic neurons (Jane Garland et al., 2016). SSRIs cause a wide range of unpleasant side effects such as nausea, dizziness, insomnia, or sexual dysfunction (Lader, 2015). One review also reported that exposure to SSRIs significantly increased the risk of suicide by approximately three-fold in children and adolescents (Barbui et al., 2009).

Hypothalamic-pituitary-adrenal (HPA) axis, serotonergic systems, and neurotrophic factors are known to be involved in the pathophysiology of depression (Moylan et al., 2013). Hyperactivity of the HPA axis, which reduces the synthesis of monoamines such as serotonin (5-HT), has been suggested as the pathophysiological mechanism of depression (Mahar et al., 2014). In addition, the level of brain-derived neurotrophic factor (BDNF) is decreased in individuals with depression (Sen et al., 2008). Decreased levels of neurotrophic factors including BDNF could contribute to the atrophy of certain limbic structures, including the hippocampus, and the prefrontal cortex, which has been observed in depression patients (Duman and Monteggia, 2006).

On the other hand, *Uwhangchungsimwon* (UCW) is a commonly used herbal medicine in Korea (Kim et al., 2008). UCW is standardized drug produced according to manufacturing guideline by Ministry of Food and Drug Safety (MFDS) of Korea, and approximately, 20 million pills of UCW have been being used annually in Korea (Financial Supervisory Service, 2011). UCW was first recorded in a traditional Chinese medicine (TCM) text book, called *Taipinghuiminhejijufang*, in 1107 and has been widely used for patients who have suffered stroke, convulsions, or unconsciousness (Cho et al., 1997). Another representative traditional Korean medicine book, *Donguibogam*, stated that UCW can treat mood disorders, such as depression (Heo, 2013). Traditional Korean medicine (TKM) has been used to treat depression as a concept of ‘*Ulbyeong*’ which means depressed mood with feelings of despair or uneasiness (Ng et al., 2006). UCW is a representative herbal drug that has been used to treat ‘*Ulbyeong*’ since ancient times in Korea.

Our previous studies found that UCW modulated the activation of the HPA axis by suppressing the release of stress hormones (corticosterone and/or adrenaline) under restraint stress and exerted a neuroprotective effect by inhibiting inflammation in hippocampal cells (Lee et al., 2014; Choi et al., 2016). *Aquilariae Lignum*, a main compositional herb in UCW, regulated the in microglial cell activation under condition of lipopolysaccharide treatment (Lee et al., 2018). Decreased hippocampal neuroplasticity and over-activation of brain microglial cells may contribute to major depressive disorder or depressive behaviors (Malykhin and Coupland, 2015; Stein et al., 2017). These findings let us hypothesize that UCW can be used as an antidepressant in clinic. However, no experimental evidence on the anti-depressive effects of UCW has been revealed to date.

We herein investigated the anti-depressive properties of UCW and its underlying mechanisms using a social isolation stress-induced depression-like mouse model.

## Materials and Methods

### UCW Preparation

UCW was obtained from Kyoung-Bang Pharmacy (Incheon, South Korea, Lot. No. PLP 186-2017-7002). Briefly, all drugs used in this formulation abided by Korean Pharmacopoeia standards. The components of UCW were prepared together as a superfine powder, mixed with Mel (honey), and formed into pills (3.75 g total weight). The resulting pills were individually wrapped with a gold foil. UCW was prepared based on the manufacturing process of the Ministry of Food and Drug Safety (MFDS) in Korea, and the contents of each ingredient were confirmed according to the quality control guidelines of the MFDS. The dosage of UCW used in our experiment was determined based on clinical use. In the clinic, a 60 kg adult takes one pill of UCW daily. The maximum dosage (400 mg/kg per day) was determined based on our preliminary experiment and the human clinical dose (3750 mg/adult). UCW was ground into powder and dissolved in distilled water.

### Fingerprinting of UCW

Thin layer chromatography (TLC) chromatogram has been produced by a pharmaceutical company (Kyoung-Bang). The company presented it for every batch to Korean FDA for the verification. The compositional species and amounts of the 18 medicinal herbs and four animal-derived materials in UCW are listed in **Table 1**. TLC was conducted to assess the quality of 18 medicinal herbs, *Dioscorea polystachya* Turcz., *Panax ginseng* C.A Meyer, *Typha orientalis* C. Presl, *Massa Medicata Fermentata*, *Cinnamomum cassia* (L.) J. Presl, *Paeonia lactiflora* Pall., *Liriope muscari* (Decne.) L.H. Bailey, *Scutellaria baicalensis* Georgi, *Angelica gigas* Nakai, *Saposhnikovia divaricata* (Turcz.) Schischk, *Atractylodes lancea* (Thunb.) DC., *Bupleurum falcatum* L., *Platycodon grandiflorus* (Jacq.) A. DC., *Prunus armeniaca* L., *Wolfiporia extensa*, *Ligusticum officinale* (Makino) Kitag., and *Zingiber officinale* Roscoe, respectively.

**Table 1:** Composition of UCW.

Herbal name	Scientific name	Place of origin	Amount
***Herbs***			
Dioscoreae Rhizoma	*Dioscorea polystachya* Turcz.	South Korea (Yeongju)	282 mg
Glycyrrhizae Radix	*Glycyrrhiza glabra* L.	China (Nei meng gu)	202 mg
Typhae Pollen	*Typha orientalis* C.Presl	China (Hubei)	100 mg
Ginseng Radix	*Panax ginseng* C.A Mey	South Korea (Geumsan)	97 mg
Massa Medicata Fermentata		China (Fujian)	100 mg
Cinnamomi Cortex	*Cinnamomum cassia* (L.) J.Presl	China (Fujian)	70 mg
Angelicae Gigantis Radix	*Angelica gigas* Nakai	South Korea (Jeongseon)	60 mg
Atractylodis Rhizoma Alba	*Atractylodes lancea* (Thunb.) DC.	South Korea (Bonghwa)	60 mg
Paeoniae Radix Alba	*Paeonia lactiflora* Pall.	South Korea (Jeonnam)	60 mg
Scutellariae Radix	*Scutellaria baicalensis* Georgi	South Korea (Jeonnam)	60 mg
Liriopis Tuber	*Liriope muscari* (Decne.) L.H.Bailey	South Korea (Milyang)	60 mg
Saposhnikoviae Radix	*Saposhnikovia divaricata* (Turcz.) Schischk	China (Nei meng gu)	60 mg
Bupleuri Radix	*Bupleurum falcatum* L.	South Korea (Jeongseon)	50 mg
Platycodi Radix	*Platycodon grandiflorus* (Jacq.) A.DC.	South Korea (Yucheon)	50 mg
Cnidii Rhizoma	*Ligusticum officinale* (Makino) Kitag.	South Korea (Youngyang)	50 mg
Poria Cocos (Hoelen)	*Wolfiporia extensa*	South Korea (Bonghwa)	50 mg
Armeniacae Semen	*Prunus armeniaca* L.	China (zhong ya)	50 mg
Borneo Camphor	*Dryobalanops aromatica* C.F.Gaertn.	China (Guangdong)	41 mg
Zingiberis Rhizoma Crudus	*Zingiber officinale* Roscoe	South Korea (Damyang)	30 mg
***Animal derives***			
Asini Gelatinum	*Equus asinus* L.	China (Sandong)	70 mg
Calculus Bovis	*Bostaurus Linne var. domesticus* Gmelin	China (Hubei)	14 mg
Saigae Tataricae Cornu	*Saiga tatarica* L.	China (Xin jiang)	35 mg
***Single compound***	L-muscone	South Korea (Woori Chemtech)	75µg
***Diluting agents***			
Mel(honey)	*Acalypha indica* Radoszkowski	South Korea (Yechon)	1.998 mg
Aurum	Gold	South Korea (Anseong)	Quality standard
		Total	3.75 g

The quality control for *Bostaurus Linne* var. *domesticus* Gmelin and *Glycyrrhiza glabra* L. was performed using a high-performance liquid chromatography (HPLC) coupled with high resolution LTQ Orbitrap mass spectrometry (MS) system (Thermo Scientific Co., San Jose, CA, USA), and identifying peaks were quantified with each relative reference compound. Reference compounds, bilirubin (B4126) for *Bostaurus Linne* var. *domesticus* Gmelin, glycyrrhizic acid (1295888) for *Glycyrrhiza glabra* L., were purchased from Sigma (St. Louis, St. Louis, MO, USA). The analytical column with Kromasil C18 (4.6 × 250 mm particle size 5 µm) was maintained at 30°C. The mobile phase conditions contained methanol and 2% of acetic acid for *Bostaurus Linne* var. *domesticus* Gmelin (9:1), and a mixture of acetonitrile, water and phosphoric acid solution was used as the mobile phase for *Glycyrrhiza glabra* L. (35:65:0.05). The analysis was operated at a flow rate of 1.0 ml/min and observed under the UV light (436 or 254 mm). The injection volume was 10 μl.

For additional analysis of L-muscone, gas chromatography/mass selective detector (GC/MSD) was used. L-muscone was purchased from Woori Chemtech (Anseong, South Korea). 3% OV-1 on chromosorb W-HP (80–100 mesh) was used as the column (diameter 3 mm, length 4 m). The column was maintained at 180°C during the performance. Nitrogen was used as mobile phase. The velocity of flow was 45 ml/min. The injection temperature was 270°C and the injection volume was 3 μl. Quantitative analysis was analyzed simultaneously by Chemstation software (Agilent Technologies, Santa Clara, CA, USA).

### Chemicals and Reagents

The following reagents and chemicals were obtained from Sigma-Aldrich (St. Louis, MO, USA): paraformaldehyde solution, radioimmunoprecipitation assay (RIPA) buffer, sucrose, Triton X-100, skim milk powder, 4′,6-diamidino-2-phenylindole (DAPI). Normal chicken serum was purchased from Vector laboratories (Burlingame, CA, USA). The other reagents were obtained from the following vendors: 5-HT, tryptophan hydroxylase 2 (TPH2), cAMP response element-binding (CREB), phospho-CREB, BDNF, beta-actin antibodies, and horseradish peroxidase (HRP)-conjugated horseradish peroxidase secondary antibody for western blotting (Abcam, Cambridge, MA, USA; and Santa Cruz Biotechnology, Santa Cruz, CA, USA).

### Animals and Experimental Design

Forty-eight speciﬁc pathogen-free C57BL/6J male mice (six weeks old, 18–20 g) were purchased from Koatech (Gyeonggido, Republic of Korea). All mice were housed in plastic cages maintained at 24 ± 1°C with a 12 h: 12 h light-dark cycle (light on 7 am and off 7 pm). The mice were freely fed food pellets (Cargill Agri Furina, Gyeonggido, Korea) and water. After acclimation for 7 days, the mice were randomly divided into six groups (n = 8): Control, SI (social isolation), UCW treatment (100, 200, or 400 mg/kg) and fluoxetine treatment (20 mg/kg).

The isolation procedure was conducted based on previously described procedures (Ibi et al., 2008; Dang et al., 2015). Our experimental scheme is summarized in **Supplementary Figure 1**. All mice except those in the control group were isolated in each cage (26 × 18 × 13 cm) for 31 days, but the mice in the control group were housed under normal conditions with eight mice together in a same cage (26 × 18 × 13 cm). Mice were orally administered distilled water, UCW (100, 200, or 400 mg/kg), or fluoxetine (20 mg/kg) once per day for the final 17 days. Three behavioral tests, tail suspension test (TST), forced swimming test (FST), and open field test (OFT) were performed sequentially during the final 3 days. Mice were sacrificed under 8 h of fasting (from 7 am to 3 pm) and CO_2_ inhalation anesthesia one day after the open field test. In order to minimize the time-related gaps among 6 groups, a mouse from each cage was sacrificed one by one sequentially.

This study was carried out in accordance with the Guide for the Care and Use of Laboratory Animals published by the United States National Institutes of Health (NIH). The protocol was approved by the Institutional Animal Care and Use Committee (IACUC) of Daejeon University (DJUARB2017–013).

### Tail Suspension Test, Forced Swimming Test, and Open Field Test

The TST was performed following the procedure described previously (Can et al., 2012). Each mouse was individually suspended (2 cm apart) by its tail from the top of a box (30 × 30 × 50 cm) for 6 min. The test was carried out in a darkened room with minimal background noise. The duration of immobility and global activity was recorded during the final 4 min of the test.

The FST was carried out according to the method of Castagné (Castagné et al.,2011). Each mouse was forced to swim individually for 6 min in a plastic cylinder (50 cm in height and a diameter of 20 cm) containing fresh water up to a height of 23 cm at 25°C. The duration of immobility was recorded during the final 4 min of the test. In addition, the latency to immobility (defined as motionless for at least 1 second) was recorded. Three observers (under blind status) scored their judgments of the time points, and the average was used as the final point.

The OFT was performed as previously described (Ieraci and Herrera, 2006). The open field apparatus was a four-sided plastic enclosure (40 × 40 × 30 cm) with white side walls and a white floor divided into nine equal squares by black lines. Each mouse was placed in the central square of the apparatus and observed for 5 min, and the following behaviors were recorded: duration spent in center and zone transition number.

### Sample Preparation

All mice were sacrificed under ether anesthesia 24 h after the final behavioral test (OFT). Blood was collected by following IACUC criteria. Serum was collected by centrifugation at 3,000 rpm for 15 min at 4°C and then stored at -80°C. For immunofluorescence staining, after transcardial perfusion, the whole brains from three mice of each group were fixed in 4% paraformaldehyde solution. For the remaining five mice, the hippocampal region was isolated immediately from the whole brain, and then samples were stored at -80°C or in RNA later (Ambion, TX, USA). And then, the hippocampus was isolated and homogenized in RIPA buffer for biochemical analysis, such as western blotting. The protein concentrations were determined using a Bicinchoninic Acid Protein Assay Kit (Sigma) by measuring the absorbance at 560 nm using a spectrophotometer (Molecular Devices Corp., Sunnyvale, CA, USA).

### Immunofluorescence Analysis of 5-HT and the Glucocorticoid Receptor (GR)

Immunofluorescence analyses were performed using a modified method described by the previous studies (Zhang et al., 2016; Zhao et al., 2017) to observe the 5-HT and GR relative intensity in dorsal raphe nuclei (DRN) and the hippocampal CA1 area, respectively. Brain tissues were cryoprotected in 30% sucrose, embedded in tissue-freezing medium with liquid nitrogen, and cut into frozen coronal sections (35 μm) using a Leica CM3050 cryostat. Sections were stored under anti-freeze buffer. Parallel free-floating sections were treated with blocking buffer (5% normal chicken serum in PBS and 0.3% Triton X-100 for 1 h at 4°C) and incubated with primary antibodies against 5-HT (1:400, ab66047, Abcam) or GR (1:200, sc-393232, Santa Cruz) overnight at 4°C. After washing with ice-cold PBS, sections were incubated with donkey anti-goat IgG H&L (1:400, Alexa Fluor^®^ 488, ab150129, Abcam) or goat anti-mouse IgG H&L (1:400, Alexa Fluor^®^ 594, ab150116, Abcam) secondary antibodies for 2 h at 4°C. The sections were subsequently exposed to DAPI (1:1,000, D9542, Sigma) to stain cell nuclei. All immunoreactions were observed under an Axiophot microscope (Carl Zeiss, Germany). Total signal intensity was quantified using the ImageJ 1.46 version (NIH, Bethesda, MD, USA) and compared relatively with the control group.

### Determination of Corticosterone, ACTH and Catecholamines

The total serum corticosterone level was determined using a commercially available corticosterone enzyme immunoassay kit (Arbor Assays Inc., Ann Arbor, USA, catalog No. K014-H5) according to the manufacturer’s instruction. Serum adrenocorticotropic hormone (ACTH) level was determined using an MD Bioproducts ELISA (MD Bioproducts, Division of MD Biosciences, Inc., St. Paul, Minn., USA, catalog No. M046006). Dopamine, epinephrine, and norepinephrine levels in the hippocampus were measured using a commercial kit (3-CAT Research Kit, LDN, Germany), following the method described by Popović et al. (Popović et al., 2017). The absorbance was measured using a spectrophotometer (Molecular Devices) at 450 nm.

### Western Blotting Analysis

The protein activities of tryptophan hydroxylase 2 (TPH2), 5-HT, BDNF, CREB, and p-CREB proteins in the hippocampus were evaluated by western blotting, as described by Grønli et al. (Grønli et al., 2006). The proteins from homogenates were separated by 10% polyacrylamide gel electrophoresis and transferred to polyvinylidene fluoride (PVDF) membranes. After blocking in 5% skim milk, the membranes were probed overnight at 4°C with primary antibodies: Rabbit anti-TPH2 monoclonal antibody (1:1,000, ab 111828, Abcam), Goat anti-5-HT monoclonal antibody (1:1,000, ab66047, Abcam), Rabbit anti-CREB monoclonal antibody (1:1,000, ab32515, Abcam), Rabbit anti-CREB (phospho S133) monoclonal antibody (1:1,000, ab32096, Abcam), Rabbit anti-BDNF monoclonal antibody (1:1,000, ab108319, Abcam), and Goat anti-beta actin polyclonal antibody (1:1,000, ab8229, Abcam). The membranes were washed and incubated for 2 h with HRP-conjugated anti-rabbit secondary antibodies. Western blots were visualized using an enhanced chemiluminescence (ECL) advanced kit. The protein expression was semi-quantified using ImageJ (NIH).

### *in Vitro* Experiments

In order to confirm the inhibitory effects of UCW on GR, HT22 cells line (mouse hippocampal neuronal cells) and PC12 cells line (pheochromocytoma of rat adrenal medulla) were used. These cell lines were cultured in DMEM and RPMI-1640, respectively (supplemented with 10% FBS and 1% penicillin-streptomycin in both media) under conditions of 37°C and 5% CO2.

To conduct the immunofluorescent staining analysis, the PC12 cells were seeded into 60 mm dish (1 × 10^6^ cells). After incubation for 12 h, cells were pretreated with UCW (10 μg/ml) for 23 h before exposure to dexamethasone (100 nM) for 1 h. After incubation for 24 h, cells were fixed with 4% paraformaldehyde for 30 min at 4°C, and then cells were sequentially treated with GR antibody (1:50, sc-393232, Santa Cruz) and Alexa Fluor^®^ 488 goat anti-mouse (IgG) secondary antibody (1:400, ab150113, Abcam) for 1 h at room temperature, respectively. After DAPI incubation for 3 min, the fluorescence signal intensity was quantified using ImageJ (NIH).

To examine the inhibitory effects of UCW on GR nuclear translocation, the HT22 cells were seeded into 60 mm dish (2 × 10^6^ cells), and treatment conditions were equal with experimental design of PC12 cell study. The nuclear extracts of HT22 cells were separated using NE-PER^®^ Nuclear and Cytoplasmic Extraction Reagents (Thermo scientific) according to the manufacturer’s instruction. The protein expression of GR α/β isoforms (1:100, sc-393232, Santa Cruz) and Lamin B1 (1:200, sc-374015, Santa Cruz) was analyzed and semi-quantified using ImageJ (NIH). The water-solved UCW was filtered through a Whatman filter paper (Advantec^®^, Tokyo, Japan).

### Statistical Analysis

Behavioral results were expressed as the mean ± standard error of the mean (SEM), and the other results were expressed as the mean ± standard deviation (SD). The statistically significance differences between the groups were evaluated by one-way analysis of variance (ANOVA). In case significance effects for the ANOVA exist, we performed a *post hoc* Tukey’s HSD test. In all analyses, P < 0.05 was taken to indicate statistical significance.

## Results

### Quality Control of UCW

As shown in TLC analysis of UCW, all compositional herbs were verified in UCW (**Supplementary Figure 2A**). *Bostaurus Linne* var. *domesticus* Gmelin and *Glycyrrhiza glabra* L. with their dominating compounds (total bilirubin, indirect bilirubin, and glycyrrhizic acid) were analyzed using HPLC (**Supplementary Figures 2B-a–c**) while L-muscone was verified using GC analysis (**Supplementary Figure 2B-d**). The quantification of *Bostaurus Linne* var. *domesticus* Gmelin, *Glycyrrhiza glabra* L., and L-muscone are shown in **Supplementary Table 1**. These compositional analyses were approved by MFDS in Korea.

### UCW Alleviated Depressive-Like Behavior in the TST

**Figure 1:**
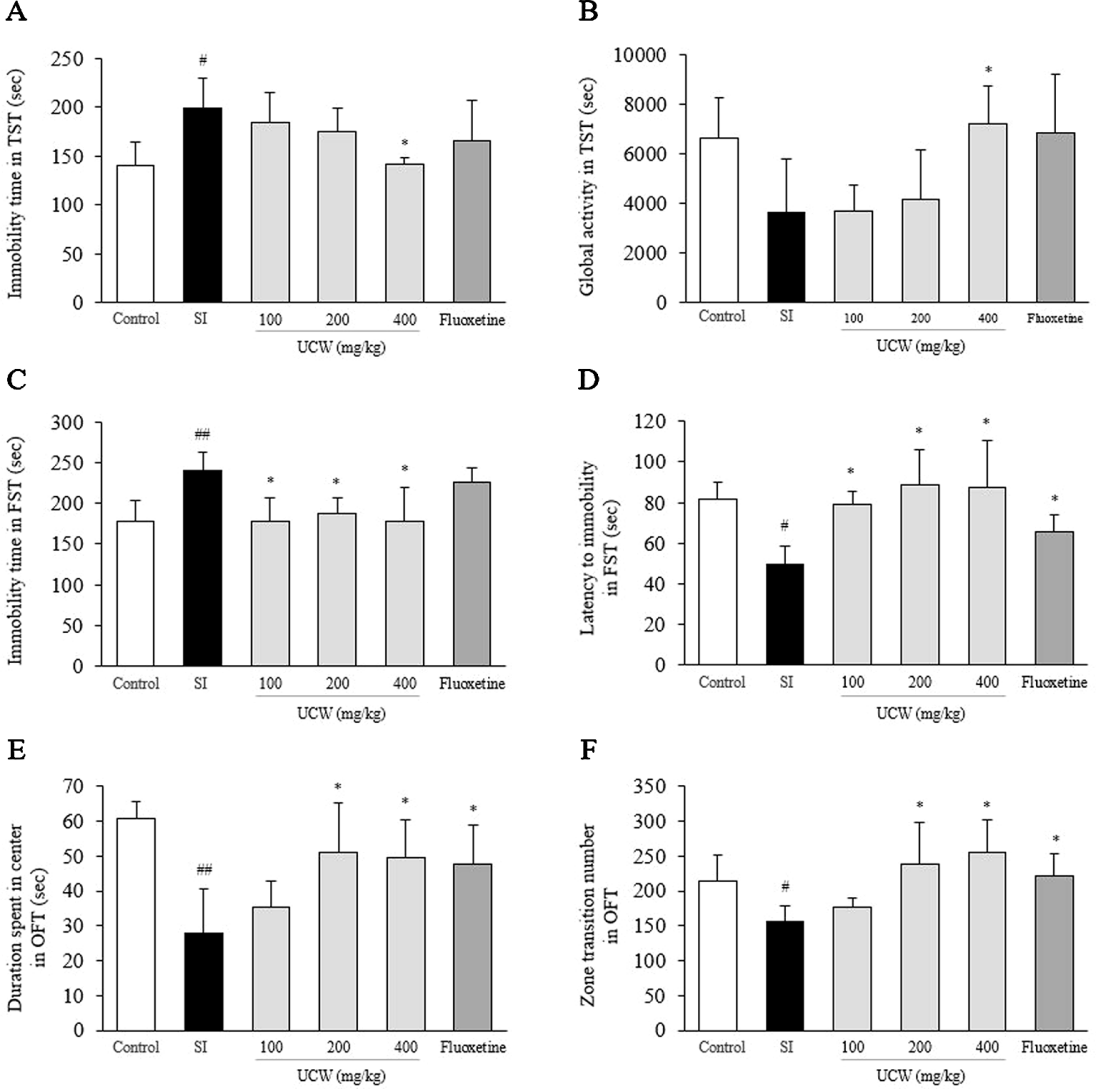
Effects of UCW in depressive-like behavioral tests. After social isolation (31 days) with/without administration of UCW or fluoxetine, the immobility time **(A)** and global activity **(B)** in the tail suspension test, the immobility time **(C)** and the latency to immobility **(D)** in the forced swimming test, and the duration spent in the center **(E)** and the zone transition number **(F)** in the open field test were measured. *Data are presented as the mean ± SEM (n = 8). ^#^P < 0.05, ^##^P < 0.01 compared with the control group; ^*^P < 0.05, ^**^P < 0.01 compared with the social isolation (SI) group.

In the TST, the social isolation stress notably increased the immobility time (1.4-fold, P < 0.05) and tended to reduce global activity (0.5-fold) compared with the control group. This behavioral change was significantly attenuated by UCW treatment (400 mg/kg) compared with the SI group for both parameters of immobility time and global activity (P < 0.05, **Figures 1A, B**). No statistical positive effect was observed for fluoxetine.

### UCW Alleviated Depressive-Like Behavior in the FST

In the FST, the social isolation stress increased the total immobility time (1.3-fold, P < 0.01) and shorten the latency to immobility (0.6-fold, P < 0.05) compared with the control group. UCW treatment, however, significantly attenuated this behavioral change in terms of the immobility time and the latency to immobility compared with the SI group (100, 200, and 400 mg/kg at P < 0.05, **Figures 1C, D**). Fluoxetine also showed the positive effect in the immobility latency compared with the SI group (P < 0.05).

### UCW Alleviated Depressive-Like Behavior in the OFT

In the OFT, the social isolation stress notably induced depressive-like behavior, as reflected by a reduction in the duration spent in the center (0.5-fold, P < 0.01) and in the zone transition number (0.7-fold, P < 0.05) compared with the control group. Treatment with UCW (200 and 400 mg/kg) ameliorated significantly these changes in depressive-like behavior compared with the SI group (P < 0.05). Fluoxetine also showed the positive effects in both duration spent in center (P < 0.05) and zone transition number (P < 0.05) compared with the SI group (**Figures 1E, F**).

### UCW Attenuated the Alterations in the Serum Corticosterone, ACTH, and Hippocampal GR Relative Intensity

**Figure 2:**
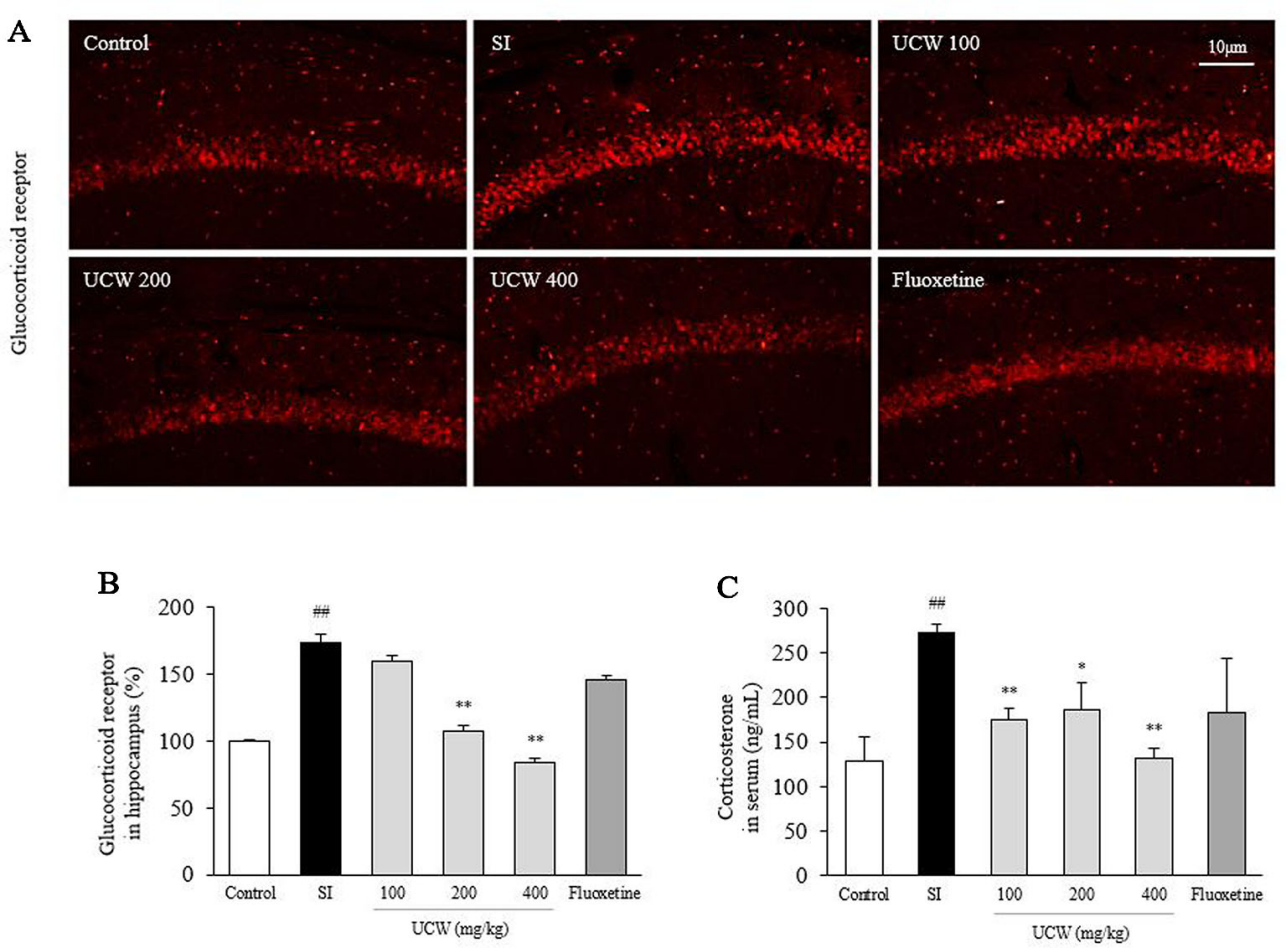
Effects of UCW on the hippocampal GR relative intensity, the serum corticosterone, and serum ACTH level. After social isolation (31 days) with/without administration of UCW or fluoxetine, the glucocorticoid receptor relative intensity was measured using immunofluorescence in the hippocampus CA1 region, and **(A)** the glucocorticoid receptor relative intensity was semi-quantified **(B)** (n = 3). The serum concentration of corticosterone **(C)** was measured (n = 8). Data are presented as the mean ± SD. ^##^P < 0.01 compared with the control group; ^*^P < 0.05, ^**^P < 0.01 compared with the social isolation (SI) group.

Social isolation stress remarkably increased both the GR relative intensity in the hippocampus (1.7-fold) and the serum concentration of corticosterone (2.2-fold) compared with the control group. UCW treatment significantly attenuated these alterations compared with the SI group; the serum corticosterone (100 and 400 mg/kg at P < 0.01, 200 mg/kg at P < 0.05), and the GR relative intensity in the hippocampus (200 and 400 mg/kg at P < 0.01), respectively (**Figures 2A, B**). Social isolation stress also notably increases serum ACTH level (2.3-fold), and then attenuated significantly by UCW treatment (100 mg/kg at P < 0.01, 400 mg/kg at P < 0.05, **Figure 5A**). No statistical positive effect was observed for fluoxetine.

### UCW Attenuates Dexamethasone-Induced GR Translocation Into Nucleus in PC12 Cells and HT22 Cells

**Figure 3:**
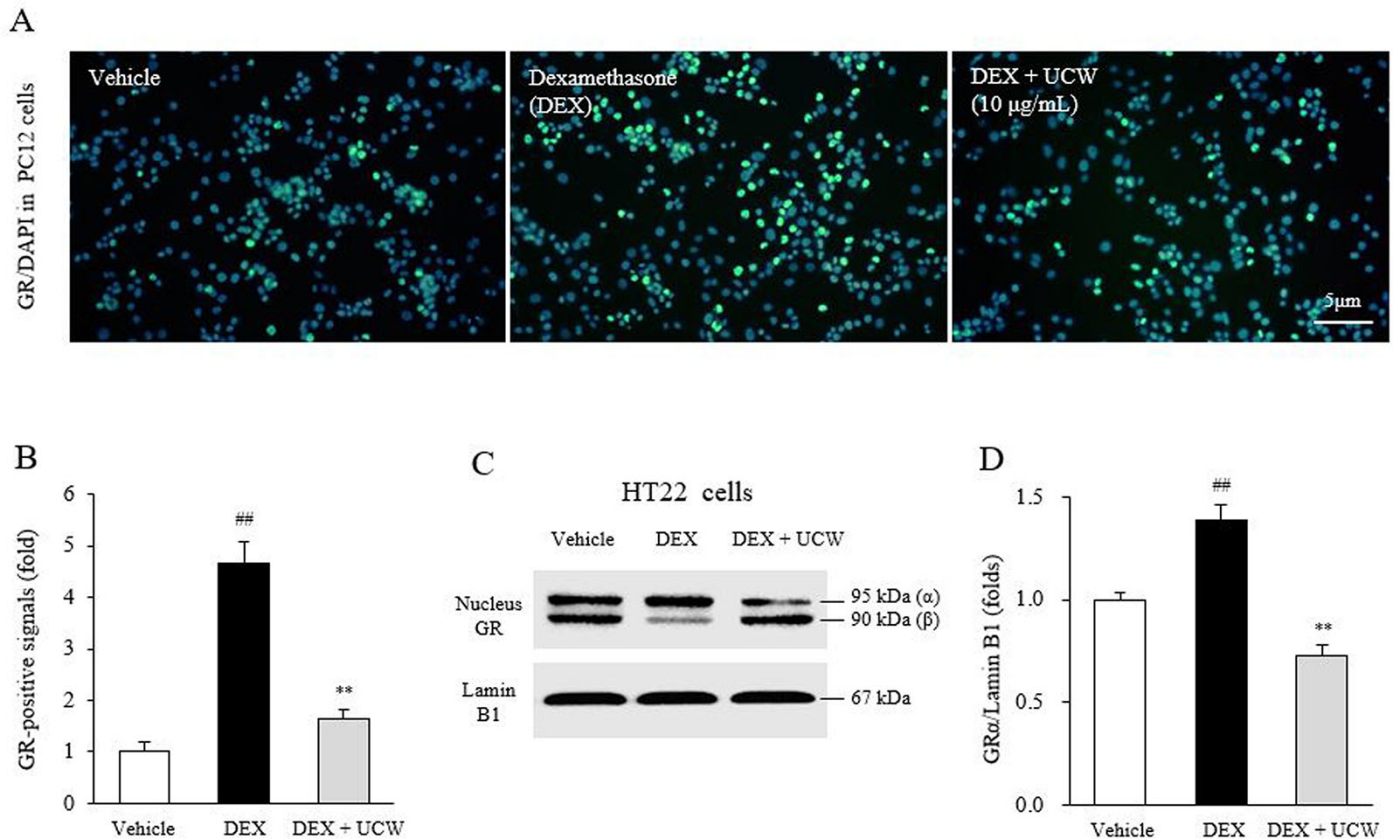
Effects of UCW on the GR in relative intensity in PC12 cells and HT22 cells. Immunofluorescence of GR in nucleus of PC12 cells **(A)** and its semi-quantity **(B)** were conducted under dexamethasone treated condition. The nuclear translocation of GRα isoform was also determined by Western blotting in HT22 cells **(C)** and its semi-quantity **(D)**. Data are presented as the mean ± SD (n = 3). ^##^P < 0.01 compared with the vehicle-treated cells; ^**^P < 0.01 compared with the dexamethasone-treated cells.

The dexamethasone treatment increased the GR-positive intensity in nucleus of PC12 cells, whereas it was significantly attenuated by treatment with UCW (P < 0.01, as shown in **Figures 3A, B**). The dexamethasone-induced GRα immunoreactivity in nucleus extracts of HT22 neuronal cells was also significantly suppressed by UCW treatment (P < 0.01, **Figures 3C, D**).

### UCW Recovered the Decrease in 5-HT Relative Intensity in Dorsal Raphe Nuclei

**Figure 4:**
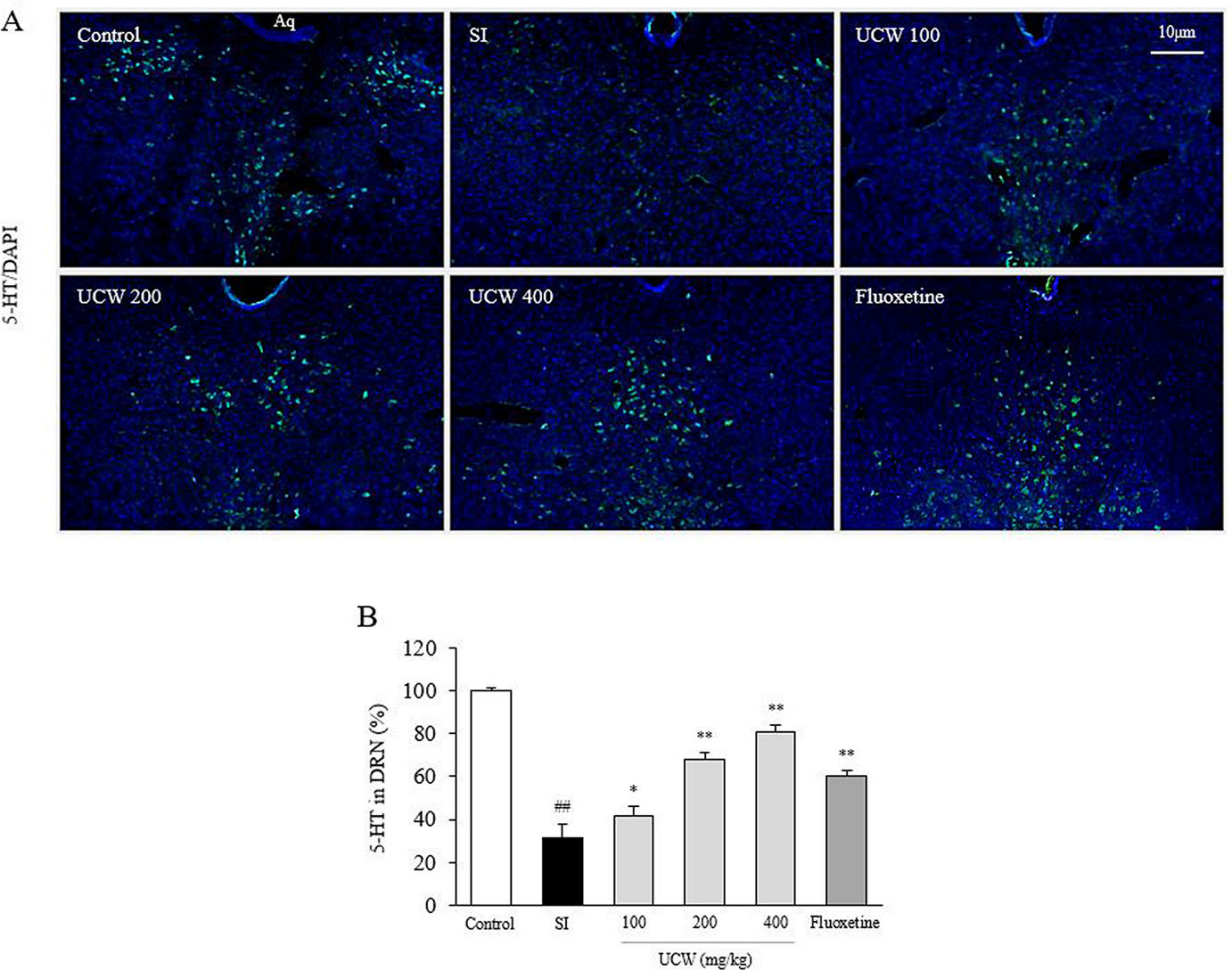
Effects of UCW on the 5-HT relative intensity in dorsal raphe nuclei. Immunofluorescence of 5-HT in dorsal raphe nuclei was measured **(A)** and semi-quantified **(B)**. Data are presented as the mean ± SD (n = 3). ^##^P < 0.01 compared with the control group; ^*^P < 0.05, ^**^P < 0.01 compared with the social isolation (SI) group.

Thirty-one days of social isolation stress drastically decreased the 5-HT relative intensity (0.3-fold) in the DRN compared with the SI group. However, treatment with UCW significantly raised the 5-HT relative intensity levels (100 mg/kg at P < 0.05, 200 and 400 mg/kg at P < 0.01) compared with the SI group (**Figures 4A, B**). Fluoxetine showed a similar effect as 200 mg/kg UCW (P < 0.01).

### UCW Regulated the Altered Concentrations of Dopamine and Norepinephrine in the Hippocampus

**Figure 5:**
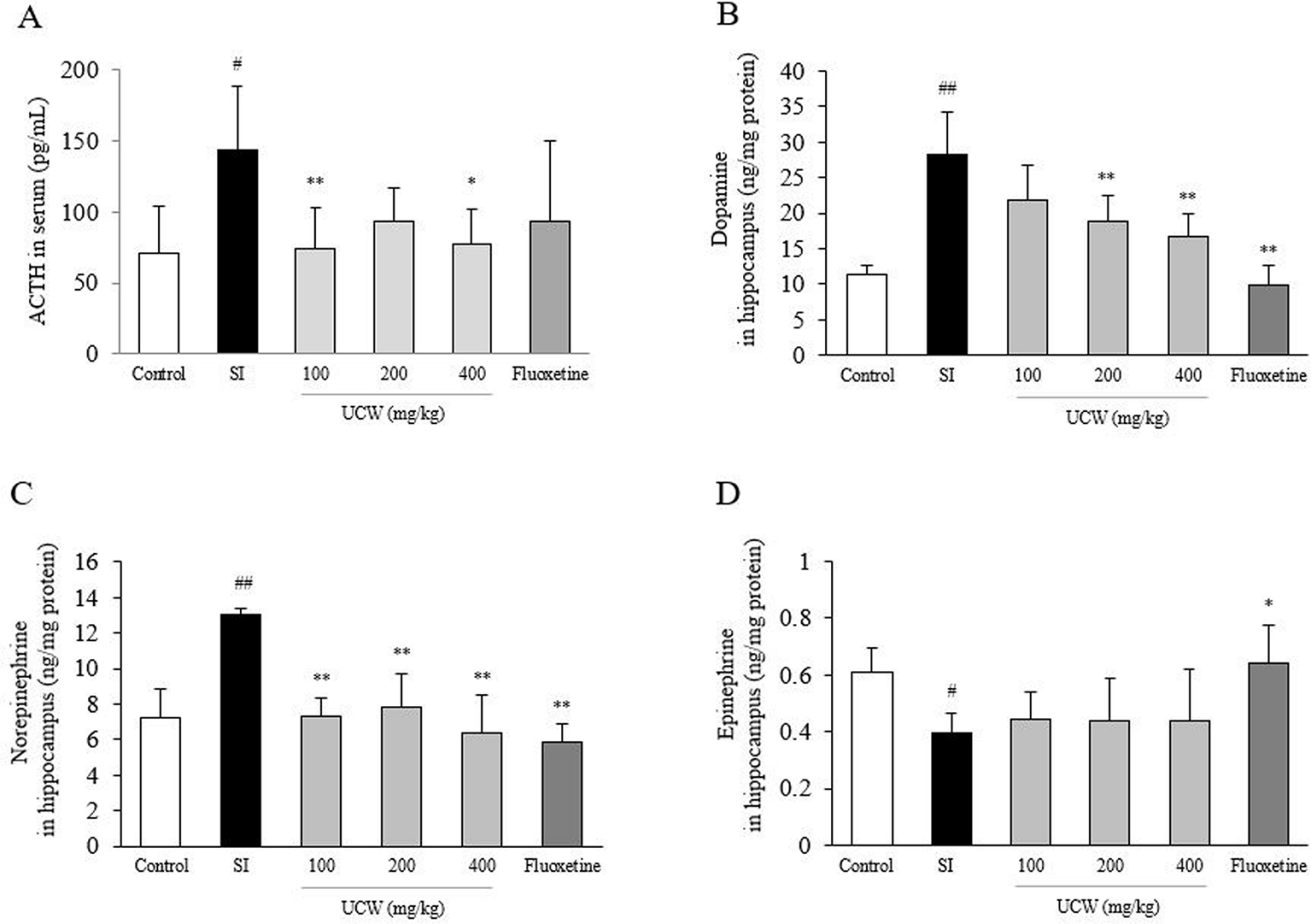
Effects of UCW on the level of serum ACTH and three catecholamines in the hippocampus. Serum level of ACTH **(A)** was measured, and then levels of dopamine **(B)**, norepinephrine **(C)**, and epinephrine **(D)** in the hippocampus were measured. Data are presented as the mean ± SD (n = 5). ^#^P < 0.05, ^##^P < 0.01 compared with the control group; ^*^P < 0.05, ^**^P < 0.01 compared with the social isolation (SI) group.

Social isolation stress dramatically changed the concentrations of three catecholamines in the hippocampal region; increases in dopamine (2.5-fold) and norepinephrine (1.9-fold), but a decrease in epinephrine (0.7-fold). UCW treatment significantly attenuated the increased levels of dopamine (200 and 400 mg/kg at P < 0.01) and norepinephrine (all doses at P < 0.01) compared with the SI group (**Figures 5B, C**). UCW treatment did not affect the epinephrine level significantly (**Figure 5D**), and fluoxetine had a positive effect on the levels of these three catecholamines (dopamine and norepinephrine at P < 0.01, epinephrine at P < 0.05, respectively).

### UCW Regulated the Decreased Activities of TPH2, 5-HT, BDNF, and CREB in the Hippocampus

**Figure 6:**
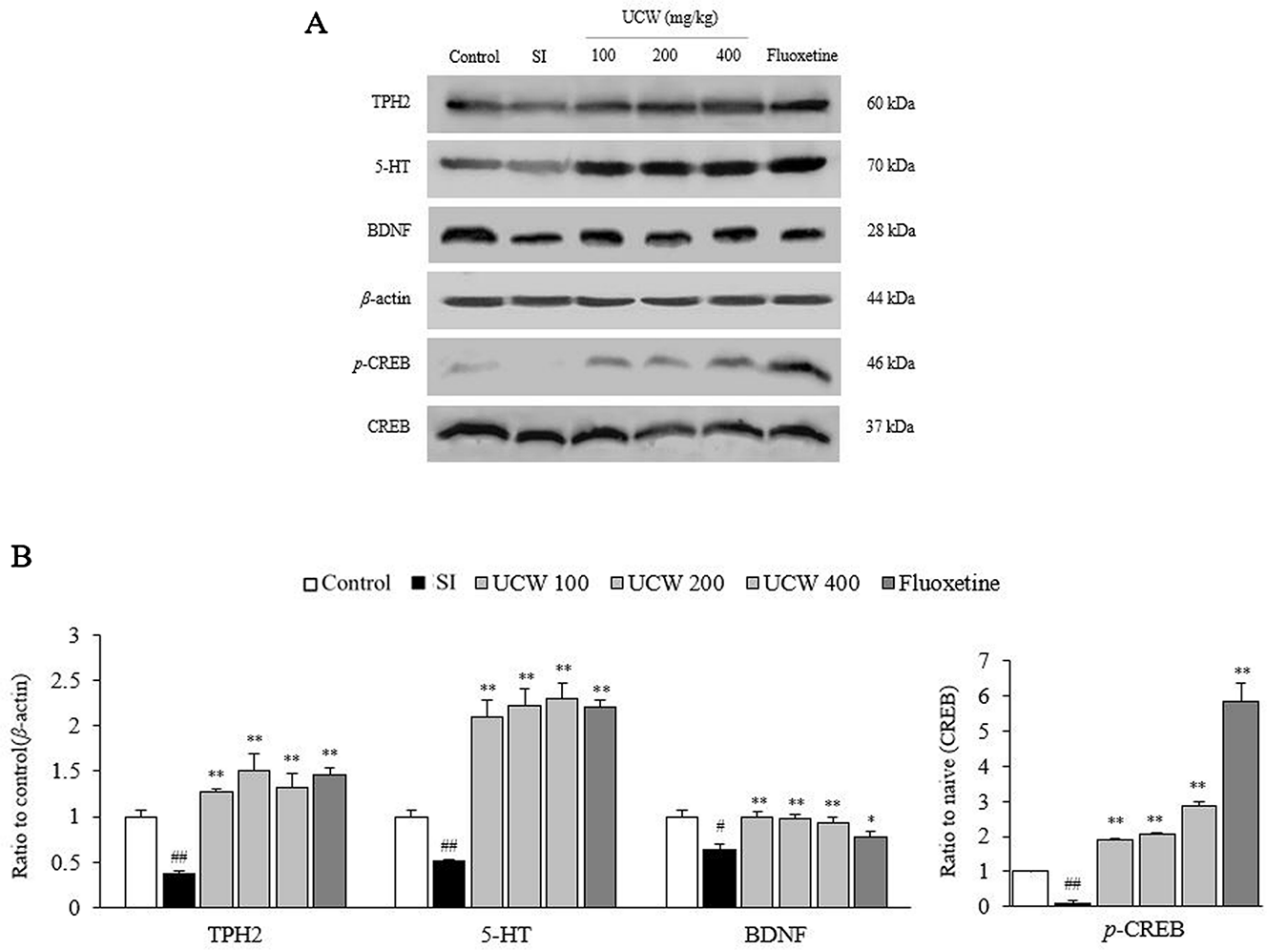
Effects of UCW on TPH2, 5-HT, *p*-CREB, BDNF in the hippocampus. The TPH2, 5-HT, *p*-CREB, BDNF levels in the hippocampus were determined by western blotting **(A)** and quantitative aliases of them were analyzed **(B)**. Data are presented as the mean ± SD (n = 5). ^#^P < 0.05, ^##^P < 0.01 compared the control group; ^*^P < 0.05, ^**^P < 0.01 compared with the social isolation (SI) group. TPH2, tryptophan hydroxylase 2; 5-HT, 5-hydroxytryptamine; BDNF, brain-derived neurotrophic factor; CREB, cAMP response element-binding protein; *p*-CREB, phosphorylated cAMP response element-binding protein.

Thirty-one days of social isolation stress drastically suppressed serotonin synthesis, as shown by measurements of the TPH2 and 5-HT protein activity (0.4-fold and 0.5-fold, respectively) in the hippocampus, but this suppression was significantly attenuated by treatment with UCW (all doses at P < 0.01). In addition, UCW treatment also recovered the isolation stress-induced depletion of the phosphorylated CREB in the hippocampus (all doses at P < 0.01). These positive effects were strongly observed in the fluoxetine group (BDNF at P < 0.05 and the rest at P < 0.01) (**Figures 6A, B**).

## Discussion

Our experimental model, involving 31 days of social isolation stress, induced typical depressive-like behavior, as shown in **Figures 1A–F**. In modernized society, social isolation is well recognized as an important factor impairing mood in humans and as one of the risk factors of depression (Cornwell and Waite, 2009). In addition, in the case of rodents, social isolation is considered a powerful stressor that causes changes in behaviors and physiological functions such as eating, learning, memory, and social connection (Beery and Kaufer, 2014). Furthermore, it is known that isolation stress causes depressive-like behaviors in rodents similar to the changes observed in humans with major depressive disorder (Filipović et al., 2017).

The TST, FST, and OFT are representative indicators for monitoring behaviors to evaluate the antidepressant effects of interventions. In these tests, mice struggle to get out of certain situations in the beginning, but as time goes by, they become inactive (Porsolt et al., 1977; Qiu et al., 2013). Consistent with previous studies, we found that depressive-like behaviors were induced by social isolation stress, but were significantly ameliorated by UCW and fluoxetine treatment (**Figures 1A–F**). These results indicated that UCW exerted an anti-depressive effect under isolation stress.

To explain the underlying mechanisms of the above behavior-derived results, we examined the changes in stress hormones induced by isolation stress. The HPA axis plays an important role in the physiology and neurobiology of the stress response and depression-relative pathogenesis (Stephens and Wand, 2012; Keller et al., 2017). Under social isolation stress, the HPA axis is hyperactivated to stimulate the synthesis and release of glucocorticoids (cortisol in human, corticosterone in rodents), which has profound effects on metabolism and behavior *via* direct actions on numerous brain regions (Nestler et al., 2002; Hawkley et al., 2012). Additionally, experimental data showed that the elevated blood concentration of corticosterone induced behavioral changes and biochemical and morphological alterations in the brain, which can be observed in depressed patients (Szymańska et al., 2009). Previous long-term follow-up study indicated that the blood ACTH was consistently higher in patients with major depressive disorder than in healthy group (Choi et al., 2018). In our study, 31 days of isolation increased both serum corticosterone level (over two-fold) and ACTH, in accordance with the GR relative intensity in the hippocampus (**Figures 2A–C** and **5A**). In addition, this effect of UCW on GR activity was supported by *in vitro* assay results using PC12 cells and HT22 cells under dexamethasone-treated condition (**Figures 3A–D**). Among two isoforms of GR, GR*α* binds to glucocorticoid responsive element (GRE) leading to gene transcription while GR*β* form inhibits this process by GR*α* (Oakley et al., 1999). The stress-stimulated HPA axis involves translocation of GR into the cell nucleus, which leads to homologous downregulation of GR and glucocorticoid resistance (Claes, 2009).

Beside learning and memory functions, it is well known that adult-generated hippocampal neurons are required for mood control and antidepressant efficacy (Eisch and Petrik, 2012). Pathological changes such as hippocampal atrophy and a reduction in hippocampal volume have been continuously reported in depressive conditions of clinical and animal studies (Boldrini et al., 2009; Duman and Duman, 2015; Chen et al., 2017). Hippocampal neuron cells, especially in the area of CA1, express high level of GRs compared to the expression in other regions of the brain and are the principal target sites for glucocorticoid-associated neuronal cell death (Tata and Anderson, 2010; Wang et al., 2013).

Repeated exposure to excessive glucocorticoids causes glucocorticoid feedback resistance and HPA hyperactivity which eventually leads to neuronal cell death (Yu et al., 2008). A majority of MDD patients (80%) showed glucocorticoid resistance and its associated cognitive impairment (Pariante and Miller, 2001). Furthermore, the altered GRs are considered a potential target of antidepressant drugs in terms of the impaired feedback regulation of the HPA axis (Anacker et al., 2011). One clinical study reported the positive effects of mifepristone, a GR antagonist (Young et al., 2004). In our results, UCW treatment significantly alleviated both the altered serum corticosterone level and GR protein expression in the hippocampus (**Figures 2A–C**).

The above anti-depressive effects of UCW and its HPA-axis-associated involvement were well supported by the immunochemical results for 5-HT in the DRN (**Figures 4A, B**). 5-HT, a monoamine neurotransmitter, is popularly thought to be a contributor to feelings of well-being and happiness; therefore, the serotonergic pathway is a main target of antidepressants, such as SSRIs (Young, 2007; Dale et al., 2016). Ninety percent of 5-HT is synthesized in the gastrointestinal track in the body, while the remaining 10% is synthesized in the brain, in particular by neurons of the DRN (Michelsen et al., 2008). As expected, we found a significant depletion of the 5-HT level in the DRN area by isolation stress but a notable restoration of this level by UCW treatment (**Figures 4A, B**). The key role of 5-HT in depression has been proposed by the finding that the synthesis of serotonin was reduced in depressed patients. Antidepressants such as tricyclic antidepressants (TCAs), SSRIs, and serotonin norepinephrine reuptake inhibitors (SNRIs) increase the levels of serotonin in the brain (Dean and Keshavan, 2017). In addition, the restoration of 5-HT by UCW treatment was confirmed by the protein analyses of 5-HT and TPH2 in the hippocampus (**Figures 6A, B**). TPH2 is the key rate-limiting enzyme of serotonin synthesis (Lifantseva et al., 2017). The levels of other monoamines such as dopamine, epinephrine, and norepinephrine are known to be reduced in patients suffering from major depressive disorders (Christensen et al., 1980; Moret and Briley, 2011). Interestingly, our animal model showed notably high levels of dopamine and norepinephrine in the hippocampus area (**Figures 5B, C**). This is a limitation of the current animal study as a depression model. The elevated levels of dopamine and norepinephrine in the hippocampus could have resulted from the stress response, which was shown in several other animal stress models (Umriukhin et al., 2007).

Hippocampal neurogenesis and plasticity are thought to be involved in mental and mood disorders (Anacker and Hen, 2017), and the BDNF is a key regulator of the formation and plasticity of neurons in brain circuits (Leal et al., 2015). The level of BDNF was found to be decreased in the hippocampus of patients with depression (Karege et al., 2005; Duman and Monteggia, 2006). Our data also showed a notable depletion of BDNF and inactivation of CREB, a key transcription factor of BDNF, in the hippocampus, which were restored by UCW treatment (**Figures 6A, B**). As the basis of the traditional concept of ‘*Ulbyeong*’ and the clinical application of UCW, previous studies showed neuroprotective and anti-neuroinflammatory effects of UCW using animal stress models (Lee et al., 2014; Choi et al., 2016). However, the BDNF-restoring evidence of UCW is first reported in the present study. Our current results may provide an antidepressant potential of UCW and its clinical relevance.

UCW is a standardized herbal drug produced according to guideline of Korean MFDS by over 20 pharmaceutical companies. Component contents of each ingredient for configuring UCW was examined for every product in comparison with major standard compounds, as indicated in the Lee’s description in 2014 (Lee et al.,2014). In the present study, we used standardized UCW that was produced by equal manufacturing process, and a typical SSRI, fluoxetine was adopted as the positive reference drug. In general, UCW (200 mg/kg) showed similar positive effects as fluoxetine (20 mg/kg). Several studies have shown that SSRIs reversed the stress-induced downregulation of BDNF in gene expression (Aydemir et al., 2005; Gervasoni et al., 2005; Gonul et al., 2005). BDNF is also known to promote the development and function of serotonergic neurons (Martinowich and Lu, 2008). In our study, fluoxetine had shown the significant effects in most of the results, but not in hippocampal GRs and serum level of corticosterone. We think that it might be caused by the inadequate dose of fluoxetine or sporadic errors in animal study.

Our present study has a limitation of unknown information about the active compounds corresponding to the anti-depressive effect. A study found that bilirubin, a major compound of Calculus Bovis in UCW, exhibits neuroprotective effects by simulating ERK1/2 phosphorylation through the nNOS/NO/cGMP system (Mancuso et al., 2008). In addition, L-muscone, a main ingredient in UCW, exerts neuroprotective effects by preventing glutamate-induced cell apoptosis *via* decreasing activation of NR1 receptor, Ca2+-CaMKII and CaMKII-dependent ASK-1/JNK/p38 signaling pathway and regulation of Bcl-2 family (Yu et al., 2014). We didn’t compare the activities of UCW with those compounds in present study model. We need further studies to identify major active compounds of UCW in the future. In addition, we need to explore the intrinsic effects of UCW under physiologic condition in the future.

Taken together, we herein provide animal-based evidence for the anti-depressive effect of UCW in social isolation stress-induced mouse model, and its underlying mechanisms may involve the regulation of the HPA axis, serotonergic system, and BDNF.

## Data Availability Statement

All datasets generated for this study are included in the article/**Supplementary Material**.

## Author Contributions

H-MO, Y-TO, and S-WK: wrote the main manuscript text, and conducted experiments. J-SL: treated mice with drugs and performed a statistical analysis and *in vitro* experiments. W-YK: prepared samples and conducted the behavioral test for tail suspension test, forced swimming test, and open field test. S-BL: conducted western blotting. Y-RC: conducted ELISA test. Y-JJ: conducted immunofluorescence staining. C-GS and J-HC: supervised the manuscript, and directed final version of all contents. All authors reviewed and approved this manuscript.

## Funding

This research was supported by the “2017 KIOM Undergraduate Research Program” funded by the Korea Institute of Oriental Medicine (grant No. C17053), and by the Ministry of Education, Science and Technology (NRF-2018R1A6A1A03025221) of Republic of Korea.

## Conflict of Interest

The authors declare that the research was conducted in the absence of any commercial or financial relationships that could be construed as a potential conflict of interest.
