# Supplementary material for: Uwhangchungsimwon, A Standardized Herbal Drug, Exerts an Anti-Depressive Effect in a Social Isolation Stress-Induced Mouse Model
Source: Front Pharmacol. 2020 Jan 31;10:1674. doi: 10.3389/fphar.2019.01674 (PMC7005224; doi:10.3389/fphar.2019.01674)
Supplement: Supplementary file 1 [file DataSheet_1.docx]

**Supplementary information**


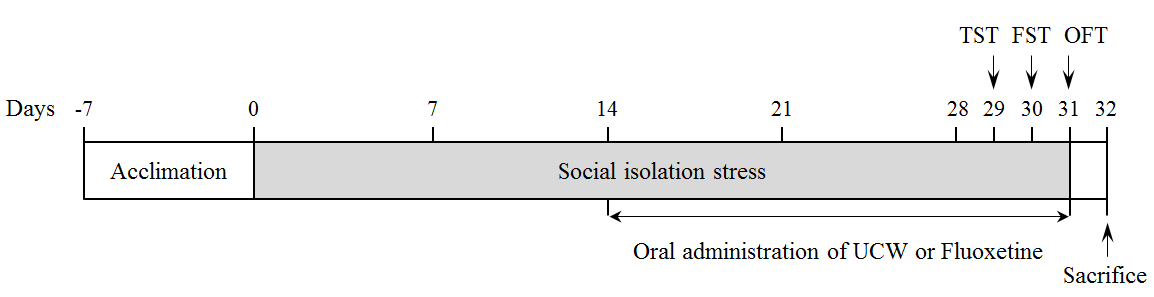


**Supplementary Figure 1. Scheme of experiment.** The experimental schedule is summarized.


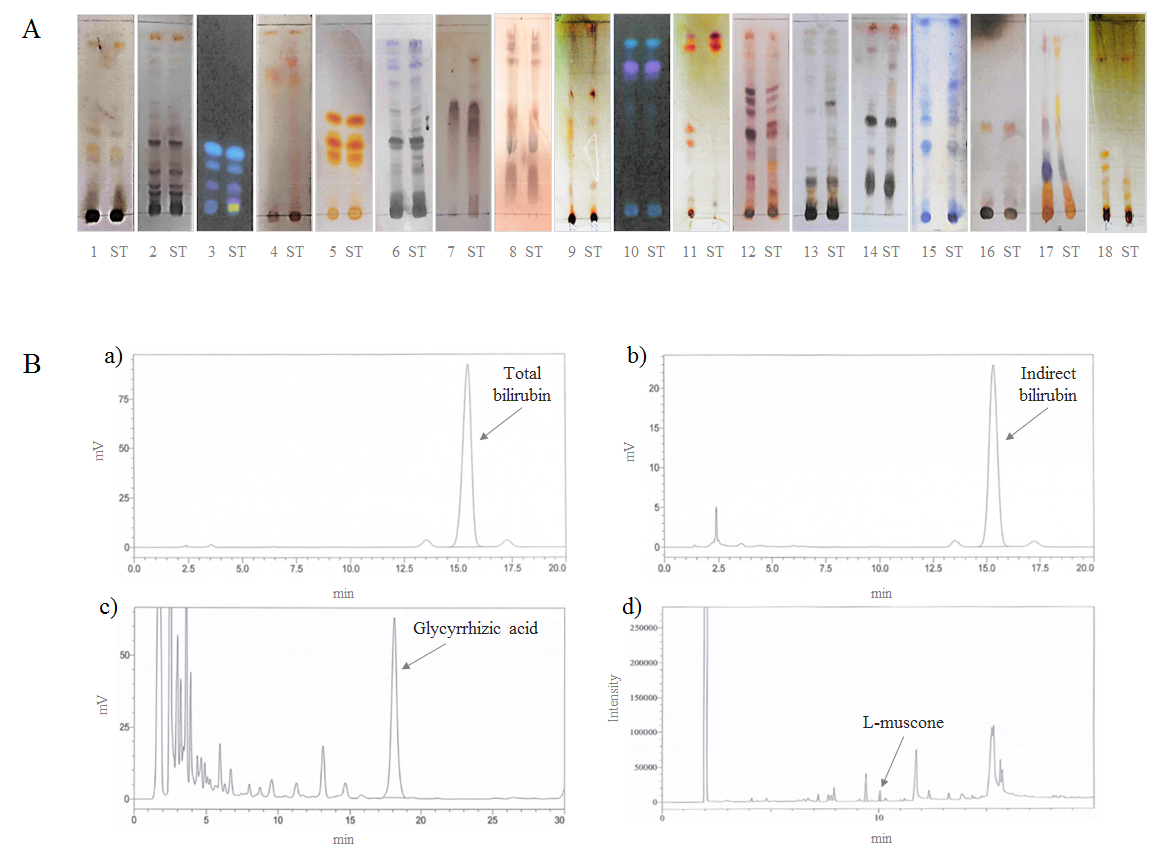


**Supplementary Figure 2. Fingerprinting analysis of UCW.**

Thin layer chromatography (TLC)-based fingerprinting was performed using CAMAG application system (Muttenz, Switzerland). Each sample was dissolved in HPLC-grade methanol and applied to prewashed silica-gel 60 F254 HPTLC plate (10 cm × 10 cm, silicagel thickness 2 mm) (Merck, Darmstadt, Germany) with an automated applicator as standard. All of the samples were separated (migration distance 75 mm) by HPLC-grade solvent hexane/acetone (1:1). The migrated components were detected after visualization with 4% vanillin sulfuric acid, under white light using Reprostar 3 with a digital camera (CAMAG). WinCATs and VideoScan software were used for data capture and analysis. Analysis of TLC identification test was conducted for 18 herbs. 1: *Dioscoreae* Rhizoma, 2: *Ginseng* Radix, 3: *Typhae* Pollen, 4: *Massa Medicata* Fermentata, 5: *Cinnamomi* Cortex, 6: *Paeoniae* Radix Alba, 7: *Liriopis* Tuber, 8: *Scutellariae* Radix, 9: *Angelicae Gigantis* Radix, 10: *Saposhnikoviae* Radix, 11: *Atractylodis* Rhizoma Alba, 12: *Bupleuri* Radix, 13: *Platycodi* Radix, 14: *Armeniacae* Semen, 15: *Poria* Cocos (Hoelen), 16: *Cnidii* Rhizoma, 17: *Borneo* Camphor, and 18: *Zingiberis* Rhizoma Crudus, respectively. Each compositional herb in UCW and its representative standard herb (ST) received from Ministry of Food and Drug Safety (MFDS) in Korea were compared (A). HPLC (a, b, c) and GC (d) analysis were performed for three materials (B).

**Supplementary Table 1. The quantification of Calculus Bovis, Glycyrrhizae Radix, and L-muscone**

| Herbal name | Scientific name | Ingredient | Content (mg/pill) |
| --- | --- | --- | --- |
| Calculus Bovis | *Bostaurus Linne var. domesticus* Gmelin | Total Bilirubin | 5.98 |
|  |  | Indirect Bilirubin | 1.45 |
| Glycyrrhizae Radix | *Glycyrrhiza glabra* L. | Glycyrrhizic acid | 28.5 |
| Active compound in male musk deer ventral glands | | L-muscone | 0.095 |
